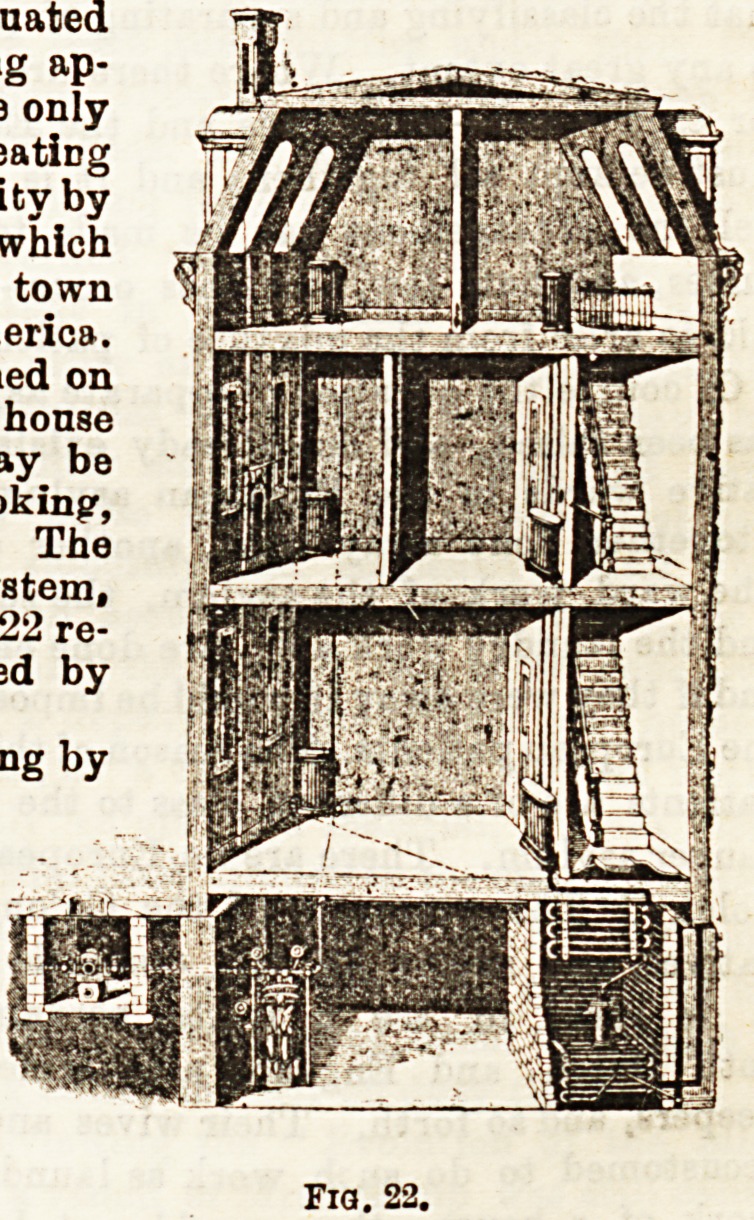# "The Hospital" Nursing Mirror

**Published:** 1896-06-13

**Authors:** 


"The Hospital" Nursing Mirror.

Being the Extra Nursing Supplement of "The Hospital" Newspaper.

[Contributions for this Supplement should be addressed to the Editor, The Hospital, 428, Strand, London, W.C., and should have the word "Nursing" plainly written in left-hand top corner of the envelope.]

### News from the Nursing World.

### A NEW HOSPITAL AT TILBURY.

On June 20th a new cottage hospital for the Tilbury and Grays District will be opened by Mr. Passmore Edwards, for the building of which he has given £2,000. The site was given by the East and West India Dock Company, £500 has been contributed by the London and India Docks Joint Committee, and Mr. Edmund Hughes (manager and director of the Tilbury Lighterage Company) is president, has given £100, and promised £50 a year for five years. The Hon. Sydney Holland is chairman, Mr. R. C. Norris acting chairman. At any hospital with which Mr. Holland has to do the nursing arrangements receive a very full share of attention, and the staff (the hospital contains eight beds) will consist of matron and two trained nurses, one for night duty and one for day, so that in the matron's absence a competent substitute will be always in charge. Miss Watson, Sister of Currie Ward at the Poplar Hospital, has been appointed to the post of Matron at a salary which will enable her to continue a member of the Royal National Pension Fund. The nurses will have in addition to their salaries half their premiums to the fund paid for them. It is very pleasant to hear of the willing personal service contributed in various quarters for the good of the little hospital. The electric light has been put into the building and the work of instalment done, as a gift, in his own time, by a man in the employ of the Dock Company. The men in the district for one week gave up sixpence in the pound to pay for the road up to the hospital, and the London and Tilbury Railway Company have given free passes to and from London for the nurses, the Tilbury Lighterage Company doing the same on their barges to and from London and out to sea.

### TORQUAY NURSE INSTITUTION.

At the annual meeting of the subscribers to the Torquay Nurse Institution the chairman directed especial attention to the small sum—£34 7s. 10d.—raised during the past year on behalf of the funds by offertories in the local churches, and suggested that he thought the clergy should realise more the great need there was of extending liberal support to the institution. It seems too often to happen that charities which lie at the very door, so to speak, become disregarded in favour of other and more sensational objects, such as foreign missions, of which stirring accounts can be given by enthusiastic preachers, and for which large collections are made; but first and foremost should be, in every town, the care of its own sick poor. It is under this head that the district nurse associations come in. The congregations of the Torquay churches, which must number amongst them many wealthy people, ought to feel heartily ashamed of their meagre contributions towards the district nursing work in the town. The nurses record more visits in the last year than ever before—23,390—and eloquent testimony to the "uniform excellence of the nursing staff" was borne by many present at the recent meeting.

### MEDICAL MISSIONS.

The energetic workers who carry on medical missions in foreign lands have many and great difficulties to contend with. The Church Missionary Society has established branches all over the world, and the doctors and nurses attached thereto unfold tales in their annual letters that may well make people who sit at home admire the pluck and determination which keeps these men and women working against opposition, ill health, and bad climates, and lacking the appliances needed for ministering to the bodily ills of their patients and converts. The quarterly publication of the Society has some interesting accounts of the work going on in India, Egypt, and Syria, and contains illustrations of the operating-room and a ward at the Kashmir Hospital.

### DISTRICT NURSING AT MELBOURNE.

The Melbourne District Nursing Society submitted the eleventh report to its subscribers early in March. The society now employs four nurses, one for midwifery alone, and its financial state is fairly flourishing. A sister in charge of the nurses' home is likely to be appointed shortly.

### NURSING AT THE CORK FEVER HOSPITAL.

An appalling condition of affairs was revealed at the Cork Fever Hospital at a late meeting of the Guardians. Sir George St. John Colthurst brought forward a resolution that the "request of the medical staff that the Board provide extra accommodation for the nurses in the fever hospital be complied with," and proceeded to read the report of the medical staff, from which it would appear that it has lately been proposed to partition off a portion of one of the wards as a sleeping-room for nurses. The report further stated that one nurse had broken down completely and hopelessly in health; that the last night nurse had resigned for the same reason; while, of four other nurses, two contracted typhus fever, one scarlet fever, and one typhoid, and one died. The doctors considered this to be the direct result of sleeping in contaminated air, and had urged the provision of a small detached building for the nurses to sleep and have meals in, but the Board had declined to carry out the suggestion. A gentleman who proposed an amendment to Sir George Colthurst's motion remarked that "the nurses never made a complaint, and when they did he thought it would be time to consider the alterations"! adding that, if they made reforms at all, why not "do something for the unfortunate lunatics, who were sleeping two in a bed . . . and were unable to make any complaint"! Ultimately Sir George Colthurst's motion was rejected. It is to be hoped that the public of Cork will take the matter up, and see to it that this scandalous state of things shall speedily be put an end to.

### TRAINING AT CARDIFF UNION INFIRMARY.

The medical officers of the Cardiff Board of Guardians have presented a report upon the training of probationers, suggesting that for the future "candidates must not be under twenty-two years of age; that applications for courses of training be made to the superintendent of nurses; that women desirous of becoming probationers should produce testimonials of character and certificates of sound constitutional health; that the time for training should be three years; that board and uniform be supplied, and that during the three years of training probationers should receive £10 for the first year, £12 for the second year, and £16 for the third year."

### DERBY BOROUGH ASYLUM.

A few days since, the chairman of the Derby Borough Asylum Committee (Mr. Alderman Woodiwiss, J.P.) presented medals and certificates to the candidates who had successfully passed the recent examination for the nursing certificates of the Medico-Psychological Association. The examination consisted of a written paper set by the association, and a *vivâ voce* examination conducted by Dr. Macleod, of the East Riding Asylum. The candidates, twelve in number, who are required to produce certificates of over two years' training, were all successful, and several won for themselves special commendation. At the present time three-fourths of the nurses and two-thirds of the attendants at the asylum hold the Medico-Psychological certificates. Prizes were also awarded to those who had been most successful at the examination on the lectures given by the medical officers (Dr. Macphail and Dr. Moon) during last winter.

### A NEW INSTITUTION.

The opening of the new sanatorium at Sutton Coldfield, Birmingham, was celebrated the other day by a garden party. The building has been presented to the committee of the Birmingham and Midland Counties Sanatorium by the treasurer, Colonel Wilkinson, and is to be reserved for the special use of clerks and school teachers. Mrs. Hamilton, for several years matron of the Manchester Hospital for Incurables, has been appointed lady superintendent.

### WORKING HOURS FOR NURSES IN AMERICA.

Miss Nutting's "Statistical Report of Working Hours in the Nurse Training Schools of the United States," published in the May number of the *Trained Nurse*, will interest all English nurses, enabling them, as it does, to judge of the conditions under which their sisters across the Atlantic carry on their work, and to compare them with English methods and customs. Miss Nutting's inquiries have led her to the conclusion that "the working hours in the wards being but a portion of the day's work, are now in almost all hospitals too long," and that "a pupil in a training school may work harder to receive her training than a labouring man to support his wife and family," the hours varying from 60 to 105 hours per week. It would seem that a greater stress is laid upon "theory" in American training schools than in most English ones, in many cases one or two hours' daily "study" being compulsory, while classes and lectures almost invariably have to be attended in off-duty times. This is not usually the case in England, though no one will deny that, speaking generally, nurses' hours sadly need shortening here as in America. The root of the matter is reached in the report in considering nurse training schools as educational institutions; and the contention is, or course, incontrovertible that it is not for the sake of giving nurses better training that they are kept on duty so long, "but rather that the amount of service rendered to the hospital may be increased, that the working force of the institution may, for economy's sake, be kept low." "These long hours," continues the report, "frequently render it impossible for a nurse to profit by the teaching for which her services are supposed to be given, and with such long hours the teaching is merely offered as an advantage to attract applicants."

### THE "PATRON SAINT" OF NURSES.

American admiration of Florence Nightingale runs even English appreciation of her very close. The often-told story of her early life's work is dwelt upon once more in the June number of "The Trained Nurse" in an article by Dr. I. N. Love, being an address to the graduating class, Rebecca Hospital Training School, St. Louis.

### THE JOHNS HOPKINS HOSPITAL.

We have received a copy of the new regulations for the training nurses at this school, which include various alterations. A three years' course has been adopted, and for the future twelve scholarships will be awarded annually, eight of the value of one hundred dollars, and four of one hundred and twenty dollars, for the most creditable records of work and conduct. The hours on duty in the wards are to be "not less than eight"; in addition two hours daily are required to be devoted to lectures, classes, and studies. Another important step is the non-payment of pupils during their three years' training, to minimise the difficulties arising from which, no doubt the scholarships have been awarded. It will be interesting to see how this system will be found to answer.

### SHORT ITEMS.

The Duchess of Marlborough, who was accompanied by the Duke, opened last week a garden *fête* and sale of work at Frognal Park, Hampstead, promoted by the Ladies' Samaritan Society, in connection with the National Hospital for the Paralyzed and Epileptic.—A conversazione was given at St. Martin's Town Hall by the committee of the Registered Nurses' Society on Saturday, June 6th, at eight p.m.—The committee of the Cardiff Workhouse have granted a superannuation allowance of £13 per annum to Nurse Kenna.—H.R.H. the Duchess of York has consented to become patroness of Mrs. Kitto's Convalescent Home and Orphanage. The concert held recently at Stafford House in aid of the Home has realised a sum of more than £300.—An entertainment in aid of the Hampstead Hospital took place at the Vestry Hall, Haverstock Hill, on June 8th, at which an amusing travesty of "Cinderella" was performed by an amateur company.—The annual general meeting of the Gainsborough District Nursing Association was held on June 3rd. The Chairman, Mr. James Marshall, J.P., paid a high tribute to the work of Nurse Cooper and Nurse Batter.

### Hygiene: For Nurses.

By JOHN GLAISTER, M.D., F.F.P.S.G., D.P.H.Camb., Professor of Forensic Medicine and Public Health, St. Mungo's College, Glasgow, &c.

### X.—HEAT IN RELATION TO HEALTH: GAS STOVES, OIL STOVES, HOT-WATER PIPES, STEAM PIPES.

GAS STOVES.—These are the outcome of a public demand for a form of heating which is at once handy, cleanly, and comparatively cheap. We have practically tried every form, but have discarded them all. All the objections urged against coal stoves have greater force against gas stoves, not only from the point of view of efficiency, but mainly from that of health. That they are handy, cleanly, and relatively cost less than other heat sources, and that they have a limited field of usefulness, cannot be gainsaid. When, for instance, they are fitted into grate-places, which are filled with asbestos or fire-clay lumps, they constitute the least objectionable form, since they combine, to some extent, to give the radiant heat of the coal fire with the handiness and cleanliness of the gas fire. The same, however, cannot be said of those gas heaters which are only provided with a flue-pipe of a half to one inch diameter. Such are worse than useless, for they give to those who use them a false sense of security; and, by them, ventilation is practically non-existent. Flueless gas or oil stoves are absolutely harmful, unless adequate provision be made to neutralise by absorption the resultant gases of combustion. The former, which purport to do this, are still on their trial; but until some special provision be made for the absorption of carbon monoxide gas (CO), which is exceedingly poisonous and, at the same time, odourless, they cannot be deemed healthy. Acetylene, a very pungent and penetrating gas, is liable to be given off from gas fires when the Bunsen flames chance to become choked with soot, thereby preventing the necessary admixture of air and coal gas. It is very irritating to the throat and lungs. Cleaning of the burners will put matters right at once. It appears to us that the special field of usefulness of the gas-fire is that for cooking purposes. Where expedition, at any time, is necessary, and in summer especially, when kitchen fires are likely to be low, a gas-fire, or grill, which can be lighted in a moment, is not only handy, but exceedingly useful.

OIL-STOVES share the general condemnation. There is no practical difference between burning several wicks under cover of coloured glass and burning several single lamps equivalent to the same number of wicks. They equally consume the air of the room, and for equal quantities of heat, consume more oxygen than coal-gas. Besides, the products of combustion pass off into the general room atmosphere, and are harmful. Another objection to their use is the disagreeable odour which an imperfectly cleaned oil-stove is apt to create.

Whereas, then, the open grate, whether with quick or slow combustion, acts not only to warm the room and its occupants—and the walls equally with any other part—by radiant heat, but also acts as an excellent "outlet" ventilator; the same cannot be said of the ordinary close-stove, gas-fire, or oil-stove, which directly warms the air of the room, and the objects of the room unequally, and which does not act as an efficient ventilator. Besides, imperfect combustion of coal-gas or oil is liable to cause the production of certain malodorous gases, which are as harmful as they are disagreeable.

HOT-WATER PIPES.—Heating by pipes containing hot water at ordinary pressure is much more common in large public buildings, such as halls, churches, schools, hospitals, barracks, and the like, than in private dwellings. It is, however, now becoming more general in the latter. The hot pipes, which are made of cast iron, warm the air of the apartment by conduction, or rather, by convection, as applied to gases. Little objection can be offered to their use (except the absence of the stimulating rays of radiant heat) provided special means are adopted for the ventilation of the apartment. When this mode of heating obtains, great care must be exercised during severe frost to prevent the colder water in the return pipes from freezing. This is by no means unknown; and where no other provision for heating is made much discomfort results. In view, also, of possible disturbances of the water-supply, it is always advisable to have a reservoir upon which to fall back when the public supply is likely to be cut off for a period of time.

The high-pressure system of heating by water in pipes—under a pressure of three or four atmospheres, *i.e.*, 45 to 60 lbs. per square inch of surface—is also used in large buildings. The pipes which compose the system, and which form

Fig. 21.—High-pressure hot water coil for heating in-coming cold air of a room. A, entrance of cold air ducts; F, main pipe; H, J, branch pipe; G, expansion pipe.

a continuous whole, are made of wrought iron. They are much smaller in calibre than those of the ordinary system, because of the greater amount of heat which is given off from a smaller area, and, consequently, they occupy less space. The water is heated by one end of the pipe-system being placed in the furnace fire, and at the highest point of the system an expansion branch is formed to prevent explosions. The heat is distributed throughout a building by placing "coils" at different points. The diagram (fig. 21) shows such a "coil."

STEAM PIPES are rarely used, even in large buildings, for heating purposes. Spent steam may be used for heating conservatories which are situated close to steam-generating apparatus. Probably the only large experiment of heating the houses of a community by this method is that which was carried out in the town of Lockport, in America. The steam can be turned on or off at will in each house in the circuit, and may be used for heating or cooking, or as a motive power. The prime cost of the system, however, is high. Fig. 22 represents a house heated by steam-pipes.

FIG. 22.

Any system of heating by hot water or steam conveyed by pipes demands skilled attention, and this is more true of the high-pressure water, and steam, systems than of the low-pressure system. When they are installed into a building, arrangements of a valvular kind, which can be operated upon by a screw or wheel, usually exist, whereby the amount of heat required may be regulated.

### Some Aspects of Life in a South African Asylum.

By One of the Attendants.

### II.—THE GRAHAMSTOWN ASYLUM. WHERE IT DIFFERS FROM AN ENGLISH ASYLUM.

A nurse fresh from a large English asylum will be struck with many differences in the method of working a colonial asylum, and may at first be tempted to think that this is due to the lack of modern improvements. A little experience and observation, however, will convince her that some of these differences must exist, being the result of different conditions of life which are necessarily present. As the private life of individuals is materially affected by change of climate, of race, and of surroundings, so it follows that the life of a community in an institution must be similarly affected, and that a colonial asylum with every possible modern improvement can never be worked on exactly the same lines as an English asylum.

To begin with, the number of patients is proportionately much smaller, the buildings are differently planned, and the native and European races must, of course, be kept entirely separate. To take the Grahamstown Asylum as an instance. The buildings, as I said before, were originally a barracks, but many additions have been lately made. The number of female patients ranges from 80 to 100, of whom half are coloured people and half of European race.

The building is about equally divided, the two sides being similar in design. The natives have a living-room, a row of seclusions, and three dormitories overhead; the Europeans have the same on the opposite side of the building. There is no refractory ward, or convalescent ward, or epileptic ward; but the Europeans have a second living-room upstairs, which is set apart for the better class of patients, and the well behaved; and all the Europeans, male and female, have their meals in the large dining-hall, recently built, while the natives have their meals in their own wards.

These arrangements may seem defective to an English nurse, especially the mixing of refractory and epileptic patients with others; but when it is considered that the number of patients being so much smaller, the staff of nurses will not exceed at the most twelve or fourteen, it will easily be seen that the classifying and separating of patients is not possible to any great extent. Where there are only four day nurses for each ward—the native and the European—the patients must be kept all together; and it is wonderful to see how well the arrangement can be made to work by competent nurses, and how few accidents occur—which can really be said to arise from the mixture of patients.

Of course the question of separate asylums for the natives has been raised, and one already exists, but there are still native wards in the European asylums, and if they were altogether done away with, another difficulty would arise. The ward work of the asylum, the scrubbing and cleaning, and the laundry work also, are done entirely by the natives, and if they were away it would be impossible to get it done by the European patients. The reason of this is that the European patients are of a different class to the patients in an English pauper asylum. There are no European paupers in the Cape Colony. The peasantry of the Colony, so to speak, are the natives, and nearly all the domestic servants are natives; it is rare to see a white servant. The Europeans, both Dutch and English, are farmers, merchants, storekeepers, and so forth. Their wives and daughters are never accustomed to do such work as laundry work or the dirty work of a house; they would not be capable of doing it, even if they were willing, and it would not be right to make them do it. Consequently the rough work of an institution, as I have said, falls to the natives, who are used to it, and well able to do it, and for whom no other occupation is possible. If the natives were taken away it is probable that native servants would have to be engaged, at all events for scrubbing and for laundry work.

The needlework can, of course, be done by European patients, and at the Grahamstown Asylum there is a large, airy work-room, under the same roof as the new laundry, where all the garments are manufactured for the institution; this includes the clothes for the coloured women, who are very seldom expert with the needle, except in making patchwork, of which art they are "past mistresses."

It is well for an English nurse if she can succeed in picking up a smattering of the low Dutch spoken by the coloured people. Some of them speak English, and several understand it, but a good many do not, and they nearly all speak Dutch habitually. Even if an English nurse can make the native patients understand what she wants them to do, she will still lose much by not understanding them, because she will not know what their delusions are, and will only be able to guess at the nature of their insanity. It is always a drawback not to be able to converse freely with your patients. The Kafir language is hard to learn, but nearly all the Kafirs speak and understand Dutch nowadays.

Another suggestion that has been made is that of native nurses for native patients; but anyone who understands the native character at all will decidedly negative this idea. The native is still a savage with a veneer of civilisation, and if native nurses were placed in charge of native patients there would be no end of cruelty and ill-treatment. It will take many generations to train a native to a knowledge of what insanity is and how insane people should be treated. We can easily realise this if we consider how few educated English women are really competent mental nurses, and how few educated Europeans, of any class, know anything about insanity and its proper treatment.

Moreover, it would be difficult, if not impossible, to obtain a staff of native nurses, as the natives have a great horror of insane people and are terribly frightened of them, believing them, no doubt, to be possessed by devils. This also has its advantages, as the friends of the natives never trouble the doctors with any interference. As a rule, they are only too glad to get rid of their insane relatives; they rarely visit them, and never question the efficacy of the treatment or pry into the management of the asylum. If the friends and relatives of white patients were equally considerate—I might say equally sensible—asylum doctors and nurses would be delivered from what is now one of the chief sources of anxiety and worry in an anxious and worrying occupation.

### Minor Appointments.

Bootle Fever Hospital, Linacre, Liverpool.—Miss Edith M. Best has been appointed Ward Sister at this hospital. She was trained at Chelsea Infirmary, afterwards working at St. Bartholomew's Hospital, Chatham. Miss Best has also had some experience in private nursing.

### Wants and Workers.

[The attention of correspondents is directed to the fact that "Helps in Sickness and to Health" (Scientific Press, 428, Strand) will enable them promptly to find the most suitable accommodation for difficult or special cases.]

Nurse Willcox, Widney House, Knowle, Warwickshire, would like to exchange The Hospital for *Nursing Notes* or any other paper on nursing every Wednesday after publication.

Mrs. Brandreth, Dicklesburgh Rectory, Norfolk, writes: In this poor parish, where the work for the men has been very scarce during the winter, the women have been employed in making maternity bags for lending among the poor. The material is good and the clothes are well made, the price varying from £1 1s. to £1 5s. 6d., delivered free of charge by post. I shall be very glad to give full particulars and send list of contents to anyone who might wish to buy one.

### Nursing Conference at St. Martin's Town Hall.

A "NURSING CONFERENCE" was held on Wednesday, Thursday, and Friday in last week, in connection with the exhibition at St. Martin's Town Hall, in the lecture-room on the ground floor of the hall.

On Wednesday, June 3rd, the papers read were, in the morning, by Miss M. Mollett, matron of the Royal South Hants Infirmary, on "The Profession of Nursing; Past, Present, and Future," and by Miss Isla Stewart, matron of St. Bartholomew's Hospital, on "The Training School"; in the afternoon, by Miss Frances Hughes, matron of the Kensington Infirmary, on "Nursing in Metropolitan Infirmaries"; and by Miss Alice Wallich, late superintendent of the Woolwich, Plumstead, and Charlton District Nursing Association. On Thursday afternoon Mrs. Bedford Fenwick read a paper on "The Nursing of our Soldiers"; and a paper by Miss Loch, R.R.C., lady superintendent Indian Nursing Service, Punjab Command, was read by Miss Hyslop, of the T.N.S. On Friday three papers were contributed, in the morning on "Private Nursing," by Miss Edith Mathew-Lannowe; and on "County Council Lecturers" by Miss Henrietta Kenealy; in the afternoon on "The Evolution of the Midwife" by Miss Margaret Breay, late acting matron Metropolitan Hospital. The audiences averaged between twenty and thirty, except on Friday afternoon, when Miss Breay's paper brought together some fifty people. There was very little discussion on any occasion, no one among the audience appearing to have come with any idea of giving the meeting the benefit of her particular views on the various subjects.

Miss Mollett, in her paper on "The Profession of Nursing," traced its history from earliest beginnings, through the early Christian era and the Middle Ages, when "the best nursing was done from religious motives, and was regarded as a species of almsgiving and self-sacrifice," to the times when religious enthusiasm failed, "and as the nursing of the sick for hire was regarded with much disdain, it gradually became a purely mechanical drudge, whose connection with the sciences of medicine and surgery was not acknowledged because not understood." Then, "when the public was beginning to demand better nursing for the sick, and educated women were eager for some fresh career, Miss Nightingale appeared and popularised the idea of nurse training on thoroughly scientific and practical grounds, which appealed to English common sense." Miss Mollett referred to a recent correspondence in medical papers, in which some attempt had been made to undervalue the importance of modern nursing, but for herself she believed that in the modern trained nurse the public sees an article that meets a long-felt need. The modern nurse might not be perfect, but she made towards perfection, and "many of the faults that have been more particularly credited to her are those common to a transition period, and will die out." Miss Mollett went on to consider the Royal British Nurses' Association, as the first attempt to organise the profession of nursing, which had for its object a far-reaching educational and organising policy. Nursing as an organised profession had reached a very critical point in its career in England; there was a great future before it if it remained true to its principles, and whilst acknowledging the paramount authority of medicine and surgery in their own sphere did not forget its own professional existence and its corporate interest in all things concerning itself alone.

Miss Isla Stewart dealt with the training school of the present day, and urged the importance of a proper selection in this respect on the part of women who aspired to be nurses. Frequently their object seemed to be not to obtain the best training, but to gain a certificate for the shortest period, and to get as much time off duty as possible. In the selection of candidates there were three important qualifications—health, intelligence, and earnestness. Miss Stewart strongly approved of preliminary training for candidates, as carried out in Glasgow by Mrs. Strong, and at the London Hospital by Miss Lückes, and advocated a more thorough "weeding out" of a hospital staff by a three months' trial of probationers. A far more careful selection of the sisters, to whom the training of the probationers falls, should be the rule. Before promotion to a sister's post some training in housekeeping and in administration was most desirable, such experience to follow after a three years' course in the wards. It would also be well to weed out those probationers who were not deemed satisfactory after their first year's work. To meet the difficulty of preliminary training in the case of small hospitals, Miss Stewart explained that there should be a college for nurses under an examining board, through which intending probationers would have to pass. She looked forward also in the future to a system of "honours" for nurses. At present there was no difference made between the best and the worst.

Mrs. Bedford Fenwick's paper criticised the present system of military nursing in England. She thought that the time had now come for an increase in the number of nursing sisters employed, and for reform in the regulations concerning orderlies. She would suggest that the best educated and most suitable recruits of the Army Medical Service Corps should be detailed for the work of nursing alone, and not be called upon to do fatigue duty. They should be given three years' thorough training in one hospital, unless required for active service, and when they left the service, should be organised into a reserve corps, ready to be called upon in the event of war.

Miss Loch contributed a description of the life of Indian nursing sisters, and of the growth of the service, originated in 1888 at the instance of Lord Roberts, when the Secretary of State for India sanctioned the sending out, as an experiment, of 12 lady nurses. In 1890-91 18 new sisters were sent out, and the total strength of the service now reached 52. At first the sisters were intended to carry on only a work of superintendence, but it soon became apparent that if they were to teach the orderlies under them they must work with them, showing them how to wash the patients and make their beds. The native ward servants were of little use from a nursing point of view, being incurably lazy. The sisters worked very hard, and English nurses must not imagine that in the Indian Nursing Service they would find the order and regularity of hospital life; there were many difficulties to contend with. They had sometimes to work under medical officers who had never worked with a lady nurse before, and it was most hard to organise matters smoothly and efficiently. And when perhaps this had been accomplished, there would be a change in the staff and the ground had to be gone over again. The post of a deputy superintendent was no easy one to fill. There was, as a rule, not much surgical work; the cases were mostly enteric and malarial, cholera being comparatively rare. Miss Loch alluded to the disappointment felt in the service that some of the sisters were not sent more to the front in the Chitral Expedition, and concluded her paper with a few words on the pleasant social life which is possible to the nursing sisters in their off duty times.

Miss Breay, in reviewing the "Evolution of the Midwife," remarked that a vision of Mrs. Gamp was still called up by mention of the word "midwife." Hospitals had so reformed that her name was no longer associated with their nurses, but with the midwife it was otherwise. She was still where Dickens found her. Miss Breay held that every nurse training school should include in its curriculum three months' training in monthly nursing, and that it was undesirable that any woman who had received no general training should be allowed to train only as a midwife. Miss Breay's contention was in fact that every midwife should be a trained nurse, and every trained nurse a midwife. The London Obstetrical Society had done a valuable work in aiming at a definite standard, and its diploma far exceeded in value the certificate of any school. The qualification of three months' midwifery training, should, Miss Breay considered, be a requirement of registration.

A short discussion followed, in the course of which Mrs. Greenwood pointed out the need for a class of women, having a certain amount of training, who could yet attend at a low fee those poor mothers who now were left in many instances to most ignorant women. Three or six months' training would greatly minimise the amount of suffering at present caused through ignorant so-called midwives.

### Midwifery Papers.

### III.—SIGNS AND SYMPTOMS OF PREGNANCY.

### Diameters (Fœtal Head).

The size of the fœtal head bears an important relation to the size of the maternal pelvis through which it passes. The most important diameters of the fœtal head are: (1) Occipito-frontal, 4½-4¾ inches from the occipital protuberance to the centre of forehead; (2) occipito-mental, 5-5¾ inches from the occipital protuberance to the point of the chin; (3) bi-parietal, 3¼-3¾ inches from the protuberance of one parietal bone to that of the other; (4) bi-temporal, 3¼-3½ inches taken from one temple to the other. When opportunity occurs, it is extremely interesting to confirm these diameters on the fœtal skull and compare them with the diameters of a skeleton female pelvis.

### External Signs and Symptoms of Pregnancy.

The most noticeable are: (1) Cessations of menses; (2) morning sickness; (3) mammary changes; (4) enlargement of uterus and abdomen; (5) fœtal movements; (6) fœtal heart sounds; (7) intermittent uterine contractions and uterine souffle; and (8) violet-colour of cervix and vagina.

One of the earliest symptoms of pregnancy is the stoppage of the monthly period. Though this is an important sign it cannot always be relied upon, as pregnant women have been known to menstruate during the whole nine months of pregnancy. In others the cessation of the menses may be a temporary condition of ill-health and not of pregnancy, but in conjunction with the presence of other symptoms this may be taken as an important aid in detecting pregnancy.

Morning sickness is generally troublesome in the second month, and then passes away. In highly nervous or delicate women it sometimes persists through the whole period of pregnancy, and becomes a serious complication. If the simple remedies of rest and careful dieting are not successful a doctor should be called in, as great exhaustion, with equal danger to mother and child, may result if the sickness continues for some time.

Mammary changes are more marked in first pregnancies, and generally commence about the second month. There is a sense of fulness and tenderness in the breasts, which begin to enlarge and harden; the nipples become more prominent, and the areola surrounding them spreads and darkens in pigment. In the fifth month distinct raised white spots appear on the outer edge of the dark pigmentation; this is called the "secondary areola." Milk can be squeezed from the nipples sometimes early in pregnancy.

The increase of size in the uterus is not noticed until the fundus rises, in the middle of the fourth month, above the bony girdle of the pelvis. Then, for the first time, the movements of the fœtus are felt by the mother, and this is known as "quickening." The uterus steadily rises in the abdomen, reaching the level of the umbilicus about the sixth month and ascending to immediately beneath the diaphragm in the eighth and ninth months. A fortnight before labour the uterus sinks lower in the pelvis, relieving the tension of the abdominal muscles and the pressure on the diaphragm; the mother breathes more easily, and feels altogether lighter and more comfortable. This condition is termed "lightening." The height of the uterus in the abdomen is an aid in determining the progress of pregnancy and in fixing the date of delivery.

By palpation fœtal movements can be felt through the abdominal walls. After the fifth month fœtal heart sounds can be heard by applying a stethoscope to the abdomen. The fœtal heart-beat is very rapid (about 120 to 130 beats a minute), and can be easily distinguished on that account from the mother's pulse, which is, of course, much slower. The uterine souffle is synchronous with the mother's pulse, and a soft-blowing murmur set up by the increased blood pressure in the uterus. Intermittent uterine contractions can be felt when the uterus is sufficiently high in the abdomen—generally about the fifth month. The changes in the cervix and vagina have already been noticed, and are valuable as an aid in diagnosing pregnancy.

The duration of pregnancy is about 280 days, and is generally calculated from the last day of the last menstrual period.

Pregnant women often suffer a good deal of inconvenience, especially in the later months, from the pressure of the enlarging uterus on the adjacent parts. Pressure on the bladder causes a frequent desire to micturate, and occasionally incontinence. Constipation and piles often result from the pressure on the rectum and its blood-vessels, though a general derangement of the digestive system often occurs in pregnancy. The bowels should receive daily attention, and a gentle aperient taken or an enema of soap and water or olive oil given if the constipation continues. The patient should be warned not to take too strong an aperient or to strain at stool, so as to avoid any danger of miscarriage. Piles are relieved by hot fomentations and an application of cold cream or coca butter. Œdema of the feet and legs, and occasionally of the external genitals, is caused by pressure on the nerves and blood-vessels, and relieved by the patient assuming the recumbent position and by the support of an abdominal belt. If the veins of the legs become varicose a bandage or silk stocking should be applied. Some abnormal conditions of pregnancy are: (1) Chorea, an exceedingly dangerous complication, and the patient should be under the charge of a doctor. (2) Salivation: if the flow is so great as to greatly distress and weaken the patient a doctor should be consulted. (3) Albuminuria is another serious complication, marked by puffiness and œdema of the face and upper and lower extremities, giddiness, unequal vision, and spots before the eyes, sleeplessness, nervous irritability, sickness, and sometimes scanty and highly-coloured urine. A doctor should be at once consulted. (4) Neuralgia of more or less severity is another complication of pregnancy. (5) Leucorrhœa is occasionally present, and if the discharge is great sets up irritation of the vagina and vulva. Relief will be given by bathing the parts two or three times a day with warm Condy and water. (6) Retention of urine happens now and then in the pregnant condition. If it does not yield to the ordinary measures of hot fomentations, and if the inconvenience is great, a catheter should be passed; but this symptom should be reported to the doctor, as it may be due to more serious causes than mere pressure by the uterus.

In pregnancy a nurse should advise her patient to live as healthy and wise a life as possible; to take simple and nourishing food; to exercise daily in the open air; to wear warm, light, and loose clothing; and to avoid late hours and unusual excitement.

### A Book and its Story.

### THE CRUCIFORM MARK.

Dr. Stephens' book* is especially interesting to those who concern themselves in the unsolved mysteries surrounding hypnotism and other closely allied psychical phenomena. But though the book, as we say, specially lends itself to the mind which has given itself over to studying the truths and the untruths of the hypnotic state, "The Cruciform Mark" will prove enjoyable reading for all. There is a breeziness and a spontaneous freshness about the writing which is attractive. The local colouring is artistic, and bears the impress of reality, both during the work and the play hours of the writer. Dr. Riccardo Stephens is not an experienced or a highly-finished writer, but he is evidently a man on whom the sights and sounds of his world have made a just impression.

Written autobiographically by a medical student in Edinburgh, the volume commences with a wholly realistic scene at a post-mortem examination theatre at Edinburgh, in what Mr. Richard Tregenna, the writer, calls The Infirmary.

Though the scene is clearly brought before the reader's mind in all its details, suffice it to say that the examination is concerned over the body of a young woman, the causes of whose death appear to be wanting. The girl had been under the care of a certain medical student, by name Clegg, on whom much of the interest of the story centres. At the conclusion of the scene Clegg, the hardened habitué of the infirmary and the dissecting room, succumbs to a dead faint.

This young man, a fellow student with Tregenna, is, to all intents and purposes, the hero of the story, and the curious, baffling mystery running through the pages is closely associated with him. The autobiographer's affection for, and interest in, the other is of an infectious nature. We also become, if not Clegg's allies, interested in him. When next he makes an appearance it is at a meeting of the All Souls Club, held at Mrs. Rea-Carter's residence. The hostess, wife of an eminent biologist, is a woman who collects the young world of thought around her; she is advanced in her views, expresses them, but is withal a philanthropist. Now, at this party Clegg distinguishes himself actively in a speech in defence of the poor. Some power, not his own, Tregenna writes, "urged the fire of his words." We are given a fair hint as to the mystery in Dr. Stephens's book, when Clegg turns pale on being presented to a certain guest, a Miss Verney, whom he had not noticed as being present.

Clegg and Miss Verney had previously met, Tregenna did not know where; but the lady was reported to be seen everywhere, and was to be met by the medical students at their balls or visiting the poor. It was assumed, but not conclusively, that Miss Verney was possessed of certain occult powers; she was also attractive, and more or less of a beauty.

Some inexplicable influence is exerted by the young lady over Mr. Clegg; but his friend, Tregenna, in writing of it, does not especially rouse our curiosity, though he admits that of late Clegg had puzzled him. Restless at times, harassed at others, hilarious, too, he is a changed being. After the All Souls Club meeting, on repairing to their chambers (which the two share in common), Tregenna goes to his window and leans out, thinking over the events of the few past days. Later on, tired out, he retires to rest. From the stillness of his slumbers he was suddenly aroused by a prolonged shriek, "whether mine or another's," he writes, "I could not tell. I went to Clegg's room on the flat below; he was lying rigidly stretched on his bed, with the look of dread on his face which I had seen before; but when I woke him he only spoke of heavy supper and nightmare."

From Edinburgh, Tregenna then goes fishing to Tweedside, and charmingly is his entourage described—the exhilarating atmosphere of the hills, the still trout stream. In the midst of the peacefulness of the scene, the anglers suddenly come upon the corpse of a man evidently not long since dead. In his pocket, on a scrap torn from some pocket-book, was written: "I have wandered by the river for an hour, and the Face haunts me always. What have I done to deserve this? Some say that the dead sleep and know nothing. God grant this cannot follow me."

After several chapters, another unaccountable death further on, and then another, disarm every solution one is prepared to make. Reasoning is of no avail.

That an undefinable "something" about Clegg is answerable in some unaccountable manner, does occur to the reader; the solution was less clear to Tregenna, his friend. We turn these pages from the description of one sudden death to another—each dissimilar in its circumstances, but alike in one curious fact, that the throat of each victim was branded with a mark—a cruciform mark, hence the title of the narrative.

Tregenna, who was a personal witness to the various tragedies, after they had been enacted, which play an important part in his story, determines to solve the mystery surrounding them, and lays himself out for further investigation. His friend Clegg has, in the meantime, become of unsound mind, and the writer's entourage is one with which we feel a deep compassion; but he is to be congratulated on never for a moment giving his readers any clue to the ultimate dénouement of his tale.

And it is not until we reach the last chapter that we are given the clue to the mysteries which run through the "Cruciform Mark." What the mark was, and how it came to appear on the various victims, is not explained until the end, when the solution is reduced to a curious physical cause, which actuated unconsciously upon the unwitting enacter of many tragedies. The writer leaves us to decide for ourselves how far the book is a work of the imagination, and how far a true picture of th hypnotic power. The scientific can form but one conclusion regarding this, but judged from a literary standpoint, the "Cruciform Mark" winds up in a manner which leaves little to be regretted, and though horrors are dealt with and form the basis of the plot, yet there are fine invigorating descriptions in the pages, and a delightful freshness of expression and a breeziness straight, as it seems, from the mountain land described. The whole story is weird in the extreme, and of an exciting and absorbing nature.

* "The Cruciform Mark." By Riccardo Stephens, M.B. (London: Chatto and Windus. 1896.)

### A Brave Nurse.

Application was lately made to the Home Secretary, by special desire of Princess Christian, to recommend the Queen's bestowal of the Albert Medal upon a nurse, Miss Alford, of St. Michael's Home, Kimberley; but the proposal was not carried out. Miss Alford was sent to attend a case of pneumonia in British Bechuanaland, and, arriving at her destination, found, not only the expected patient, but a severe outbreak of small-pox, and, alone and unaided, she pluckily nursed two hundred natives and twenty white people through the disease, only losing three patients. Deeds of this kind are not done for reward, but, nevertheless, it is only right that heroism should be appreciated wherever it is found and it seems a pity that Miss Alford's devotion should go unrecognised because it took place under circumstances less thrilling than those which attend a military campaign.

### An Experience in the London Hospital.

(By a Swiss Patient.)

Why the majority of people have such a horror of the hospital is impossible to understand. Perhaps a few words from a patient who has spent thirty-six days at the London Hospital, Whitechapel, E., may do a little towards removing this great dread of entering a hospital.

I found myself, for the first time in my life, a patient in a hospital, owing to a lengthy illness, said to be incurable without an operation. I was compelled to go to the London Hospital for the operation. Before I went I made inquiries among some of my friends. Alas! little comfort did I get. My friends could only express their pity for me. Now, no words would have been considerably better; and I set out with a heavy heart, expecting ill-treatment and bad food.

I will now shortly describe my experience during my stay at the hospital. On being admitted I had a bath and was soon in bed. The nurse in the ward asked me several questions regarding my belongings. At twelve o'clock she brought dinner for the others, but she could not give me any, as the house surgeon had not seen me, and ordered my diet. Nurse came and told me she was sorry for me, because I had to look on while the others had their dinners. After the house surgeon had seen me I was told to go to the operation ward, as mine was a special case. As my operation did not take place for several days I had plenty of time to consider my circumstances, and through the kindness of sister and nurses, be it said, all my fear was taken away. When I recovered consciousness after the operation I was in my bed quite comfortable under the circumstances. As I had to lay on my back for three weeks without moving I dreaded the dreary days and nights, but my kind nurses did their best to make me happy and comfortable. Some ladies came to see the patients, bringing us buttonholes regularly every week. None but sufferers themselves can appreciate and understand such kindness. The Sunday before Christmas I was able to get up, and as Christmas is a day of great rejoicings in all the hospitals, I think a small description of our Christmas Day would not be out of place. For two days doctors, students, sisters, and nurses all worked hard, as all the wards are decorated, each nurse doing her best to make the ward under her care look as beautiful as possible, and I can truly say that the whole hospital looked like a large flower garden. The Christmas day began soon after four o'clock a.m., when we were awakened by the most beautiful singing of about fifty sisters and nurses, who went through all the wards singing Christmas carols. Father Christmas plays a prominent *rôle*, as he distributes the Christmas presents to all the patients, and I am sure it must have been hard work for him to give the 650 patients a present each. Each man had also a pipe and half an ounce of tobacco given him, Christmas Day being the only day in the year when smoking is allowed within the walls of the hospital. This privilege is greatly taken advantage of by the patients. After tea the entertainments begin, and I can say with confidence that everyone connected with the hospital did his or her best to make the patients happy, and a very happy time we had. I could not wish for a happier, and I am sure that this Christmas in the hospital will never be forgotten by any patient present. The doctors, their wives, the students and their friends did all they could to make us forget we were away from home and friends. Later on, when I had permission to go over the hospital, I had many talks with the patients, and they one and all told me of their kind treatment by the sisters and nurses. Although it is the nurses' work to look after us, every patient ought to do his or her uttermost to make their work easy. As regards the food, I can only say it was perfection and we had the very best of everything. In conclusion I can only give this advice: Let every patient make up their minds to do what nurse, sister, or doctor wants them to do, and no patient will ever regret the days he has spent in a hospital. I shall never forget what has been done for me in an institution where pain and sorrow are made easier and more bearable by the great care and kindness of sister and the nurses. May these few lines give encouragement to those who fear the hospital.

Had I had the means I would have had to pay a very large amount of money (200 guineas).

### Where to Go.

Cancer Hospital, Fulham Road, S.W.—A garden party will be held in the grounds of the hospital on Friday, June 26th, at four p.m. The band of the Royal Horse Guards (Blue) will be in attendance.

"Friedenheim" (Home of Peace for the Dying).—The annual meeting will be held on Saturday, June 20th, at half-past three p.m., in the lecture-hall of the School for the Blind (adjoining Friedenheim). The chair will be taken by Hugh M. Matheson, Esq. Tea and coffee in the grounds at half-past four.

Princess Maud's Wedding Gift. — Nurses who may not have been able to see the tea-table presented by the Pension Fund nurses to Princess Maud as a wedding gift, may like to know that until the wedding a fac-simile table can be seen at 5, New Bond Street, by the kind arrangement of Mr. Barker, the maker.

Royal British Nurses' Association.—The secretary asks us to state that the annual meeting of the association will be held at St. Bartholomew's Hospital on Wednesday, July 22. The meeting will be held at 11.30 a.m. for the appointment of scrutineers to examine the ballot papers; the ordinary business of the meeting will commence at twelve (noon). Tickets for the luncheon at St. James's Hall, price 2s. each, can now be obtained from the secretary, 17, Old Cavendish Street, W., by prepayment only. By kind permission of his Grace the Duke of Westminster, K.G., Grosvenor House will be open on that day to members of the R.B.N.A. from three to five p.m., on production of tickets, to be obtained at the offices of the association. The hon. treasurer and Mrs. Langton will be "At Home" to members of the Association at 62, Harley Street, Cavendish Square, W. Tea, four to seven p.m.

### Appointments.

St. Mary's Day Nursery and Hospital for Sick Children, Plaistow, E.—Miss E. E. Simmonds has been appointed Matron at this hospital. Miss Simmonds was trained at King's College Hospital, and for the past seven years has been a sister at the Shadwell Children's Hospital. Miss Simmonds has our best wishes for success in her new work.

Beverley Hospital and Dispensary.—Miss Alicia B. Noble has been appointed Matron of this hospital. She was trained at the Royal Hospital, Sheffield, where she also held the post of charge-nurse, and for some time acted as nurse at Beverley Hospital, afterwards taking charge-nurse's work at Fir Vale Infirmary, Sheffield. Miss Noble takes many good wishes with her to her new duties.

Liverpool Convalescent Institution, Woolton.—Miss Agnes Lumsden has been appointed to the post of Lady Superintendent at this institution. Miss Lumsden was trained at the Liverpool Royal Infirmary, where she afterwards held the appointment of assistant lady superintendent. Miss Lumsden was selected from amongst seventy-one candidates. She has our sincere wishes for her future success.

Tilbury and Grays District Cottage Hospital.—Miss Watson has been appointed Matron at this new hospital, which will be opened on June 20th. Miss Watson was trained at the London Hospital, subsequently working as staff nurse in Gloucester Ward. She has for the last year held the post of sister of Currie Ward at the Poplar Hospital for Accidents. We wish Miss Watson all success, and congratulate her upon her promotion.

Emsworth and District Jubilee Cottage Hospital.—Miss Charlotte Bray has been selected to fill the post of Matron at this institution. She was trained at the Royal Infirmary, Newcastle-on-Tyne, and has also worked at the Liverpool Hospital for Women, the Hull Sanatorium, and the Borough Hospital, Sheffield. Miss Bray was for two years head nurse of the male medical and surgical ward, Buckinghamshire General Infirmary, Aylesbury, and for two years head nurse at the Cancer Hospital, Brompton, besides having gained experience in temporary matron's duty. We congratulate Miss Bray on her testimonials, which are excellent

### Everybody's Opinion.

[Correspondence on all subjects is invited, but we cannot in any way be responsible for the opinions expressed by our correspondents. No communications can be entertained if the name and address of the correspondent is not given, or unless one side of the paper only be written on.]

### DIFFICULTIES OF A PROVINCIAL MATRON.

"ANOTHER PROVINCIAL MATRON" writes: In her letter of May 23rd "Charge Nurse" says, "Hospital etiquette asks the matron to leave the ward when the young house-surgeon stalks in." I am sure that many matrons and nurses do not so understand hospital etiquette, and would, with me, be very pleased to have the opinion of the Editor on this important point. I enclose my card.

[*** As a matter of practical working a matron would not time her visit to the ward just when the house-surgeon is going round, since presumably both the matron and the house-surgeon will desire the presence of the sister. But we never heard of a hospital where there was any rule that the matron, being a trained nurse, should leave the ward when the house-surgeon comes in, and do not think that hospital etiquette requires anything of the kind.—ED. *T.H.*]

### NURSES FOR THE POOR.

SIR,—We are anxious through your columns to make your readers acquainted with the astonishing work that has been done quietly and without ostentation by Sister Katherine (Miss Twining), in the direction of providing trained nurses for the sick poor; and to urge that it would be almost a national disgrace and loss if that work, brought as it has been to a perfection which few would have believed possible by the energy and perseverance of one woman, were now allowed to be ended for the want of a comparatively small sum of money. Struck, as Her Majesty the Queen in her kindly sympathy for her poorer subjects has also been, and as every sympathetic woman must be, with the want in the past of all proper arrangements for nursing the poor at their own homes, Sister Katherine in 1889 commenced training district and midwifery nurses at Plaistow. The work has grown beyond her means, beyond the means of her immediate friends. It has been possible for her to go on till now, but the moment is rapidly approaching when, if not helped, her life's work must end. Without giving many figures, which few would read, but which are open to all who will, the work which has been done is sufficiently impressive to justify an appeal to a wider public. In the seven years Sister Katherine has trained 182 women as midwives, all of whom have passed the London Obstetrical Society's examination, to take the place of the "old women of the village." It cannot be generally known, but it should be, that out of every 100 blind people in England, no fewer than 30 owe their calamity to the neglect by these ignorant, untrained women of the ordinary elementary rules of cleanliness. The district nurses, of whom she has trained 321, are working in 28 different countries for private people, or under county associations, so the work concerns a far larger public than the residents at Plaistow, where the nurses are trained, and where the poor benefit beyond description by their nursing. At Plaistow there are now 65 nurses being trained under proper supervision and teachers. Last year they paid nearly 100,000 visits to the sick poor in that district, and 1,200 women were attended in their confinements. The supervision, training, housing, and food, &c., of these nurses necessarily costs money. The fees paid only half meet that cost. The deficit has so far been borne by Sister Katherine and her friends. She needs now £4,000 to build a home for the nurses, it being increasingly difficult and expensive to have the nurses scattered all over the district; she needs £1,000 to pay off a mortgage and a debt; and she needs £1,500 a year to continue the work. There is no limit to the work that she and those under her can do except the limit of means. Numbers of nurses are refused each year. We ourselves know the work, we can personally vouch for its being well, carefully, and thoroughly done, and we hope the public will agree, that, having found a woman able to initiate and organise this great work, she ought to be relieved of the pressing anxiety of seeing her life's work cramped, if not ended, for the want of so comparatively small a sum. Donations may be sent to Robert Williams, Esq., treasurer of the Maternity Charity and District Nurses' Home, Howard's Road, Plaistow, E., or direct to the bankers, Messrs. Williams, Deacon, and Co., 20, Birchin Lane, E.C.—We are, your obedient servants,

WINCHILSEA, President.
MILLICENT SUTHERLAND.
MAUD SELBORNE.
EDITH WINCHILSEA.
J. W. ALBAN.
H. F. COLCHESTER.
SYDNEY HOLLAND.

### PREJUDICE IN JOURNALISM.

MISS MARGARET BREAY writes: After reading the article in last week's HOSPITAL entitled "Prejudice in Journalism," I should like to make the following comments: (1) That the statement which you quote was given considerable circulation in the daily press and allowed to remain uncontradicted; (2) that there is no one who has a greater right to speak on nursing matters than the editor of the *Nursing Record;* and that, though I am not aware that she "never tires of asserting her right to speak with authority," there is certainly no one who is better able to do so. Mrs. Bedford Fenwick, as matron of St. Bartholomew's Hospital, won for herself a world-wide reputation as an authority in nursing matters, and left the hospital at the end of six years, having gained for it a reputation second to none, and with the respect and admiration of all those who were so fortunate as to train under her. She has since that time, by her keen interest in everything that is for the good of nurses, professionally and otherwise, won the gratitude and esteem of the best class of nurses. To complain of her ignorance therefore is simply to raise a laugh at the expense of THE HOSPITAL. Lastly, we are told that "prejudice in the Press against individuals is happily rare." Perhaps—but one has only to read THE HOSPITAL weekly to find that there, at all events, it flourishes, and the present article, "Prejudice in Journalism," contains about as much spite as can well be compressed into thirty lines.

[*** We willingly print Miss Breay's praises of Mrs. Bedford Fenwick, but we hope that in what she says on this subject she is more worthy of credit than in the accusations she makes against THE HOSPITAL of indulging in "spite," for in this matter she is utterly and hopelessly in the wrong. The *Nursing Record* cannot be altogether congratulated on its champion. We drew attention to a paragraph in that paper having reference to the Chelsea Infirmary, the insertion of which in an ostensibly "nursing" journal seemed to us to show either ignorance or prejudice, but we never went so far as to say that this paragraph was copied bodily without acknowledgment from the daily press, which from Miss Breay's letter would seem to have been the case. No doubt Mrs. Bedford Fenwick will appreciate her friend's kindness in rushing to her defence, but we should think that in her editorial capacity she must wish that her champion had let things alone.—ED. *T.H.*]

### Zenana Bible and Medical Mission.

IT is generally stated by those who are closely connected with them that figures are quite inadequate to represent the work done by missionary societies. The following facts and figures, however, tell their own story. The society has working among the zenanas in India at the present time 122 European missionaries and assistants, 79 Bible women and 175 native Christians who act as teachers, nurses, &c. These missionaries and Bible women have access to 9,164 zenanas and houses, and have 2,591 regular pupils under instruction. Seventy schools have been established in connection with the society, at which there are now 3,013 pupils. Another, and not the least important branch of the work of this society, is the hospital work among the women of India. At the present time the society has three hospitals, situated at Benares, Lucknow, and Patna, which in the year 1895 treated 20,092 in and out patients, and cost £2,391 to keep up. The total income of the society raised at home from all sources amounted in 1895 to £17,161, in addition to which Rs. 46,000 was paid into the treasury in India, making the total receipts nearly £20,000. The expenditure under all heads amounted to £19,456. During 1896 a further £2,000 a year at least will be required in order to carry on the same work as last year.

### Reading to the Sick.

### LIFE'S DISCIPLINE.

**Motto.**

He that can walk under the heaviest weight without staggering, he is the strong man.—*Carlyle.*

**Verses.**

Not enjoyment and not sorrow
Is our destined end or way;
But to act, that each to-morrow
Finds us further than to-day.—*Longfellow.*

Oh Lord, the Pilot's part perform,
And guide and guard me through the storm;
Defend me from each threat'ning ill,
Control the waves—say, "Peace, be still."

Amidst the roaring of the sea,
My soul still hangs her hope on Thee;
Thy constant love, Thy faithful care,
Is all that saves me from despair.—*Cowper.*

Lord, make these faithless hearts of ours
Such lessons learn from birds and flowers;
Make them from self to cease.
Leave all things to a Father's Will,
And taste, before Him lying still,
E'en in affliction peace.
—*Hymns Ancient and Modern.*

**Reading.**

And just so it is with the higher faculties of the soul of man, with the affections of his heart; these can only be matured and grow up to their perfection in the image of Christ, through the discipline of many a struggle, and the schooling of many a grief. The path of trial and trouble is, I will not say the only path, but it is surely the ordinary path, by which a man becomes acquainted with God, or acquainted with himself. We sometimes wonder why a benevolent Father and Creator permits difficulty and suffering and slow decay to form so large a portion of the lot of His creatures. Moreover, this economy of pain is not rarely a powerful instrument in the hands of those who doubt or deny the activity of a supreme providential love. We may freely admit that the prevalence of this divine economy is, like many other things, too hard for us fully to understand; but constituted, as we are, with imperfect, though improvable faculties and affections, it would be still harder to see how it could have been otherwise with us, consistently with our moral advancement. For the world's noblest and most enduring examples come to us, for the most part, through hardship and pain; and it is difficult to see what scope there would be for the exercise and development of the finest of human qualities, such as patience, and sympathy, and bravery, and magnanimity, and forbearance, if there were no wrongs to remove or to endure, and no sufferings to assuage. Depend upon it, my brethren, love has many other phases besides joy, and wisdom has many other objects to provide for, besides a painless existence. It is said of the greatest and best man this earth has ever seen, the Divine Man, that He was a man of sorrows and acquainted with grief; and a similar measure must be meted out to His disciples, for they, too, like their Master, can only be made perfect by the things which they suffer.—*Rev. C. Pritchard, D.D.*

### Notes and Queries.

The contents of the Editor's Letter-box have now reached such unwieldy proportions that it has become necessary to establish a hard and fast rule regarding Answers to Correspondents. In future, all questions requiring replies will continue to be answered in this column without any fee. If an answer is required by letter, a fee of half-a-crown must be enclosed with the note containing the enquiry. We are always pleased to help our numerous correspondents to the fullest extent, and we can trust them to sympathise in the overwhelming amount of writing which makes the new rules a necessity. Every communication must be accompanied by the writer's name and address, otherwise it will receive no attention.

**Queries.**

(69) *Massage.*—Please tell me where I can learn massage in Manchester.—*Miss B.*

(70) *Private Nursing.*—Will you give me the names of private nursing institutions in Margate?—*A. L.*

(71) *Holidays.*—Could you suggest an economical holiday for two nurses, in September, mountain scenery preferred, not seaside, and as far as possible out of the way of excursionists? Wales or the Lakes we thought of.—*An Inquirer.*

(72) *Midwifery Training.*—I should be glad to know the address of the hospital in Edinburgh where training in midwifery is to be obtained, also the names of similar hospitals in Manchester and Liverpool.—*Newcastle.*

(73) *Supervision.*—*L. B. P.*

(74) *Nursing Abroad.*—Will you advise three nurses how to obtain appointments in a hospital abroad?—*Nurse.*

(75) *The Presentation Album.*—Will it be possible to see the album presented by the Prince of Wales to Mr. Burdett?—*H.*

(76) *Medical Advice.*—Can you recommend me a good ear specialist?—*Ipswich.*

(77) *A Brass Plate.*—I am anxious to obtain daily, hourly, or other nursing, locally. I am a fully trained and certificated nurse, and wish to fix a brass plate to my door. Will you tell me what would be correct to put on the plate and on my cards?—*Mrs. J.*

(78) *Holidays.*—You kindly offer information to holiday-making nurses. Will you tell me something about the Lake district? Another nurse and myself wish to go there for a week in July, and would be glad to know the best place to stop at.—*L. C.*

**Answers.**

(69) *Massage* (*Miss B.*)—Write to the hon. secretary of the Society of Trained Masseuses, Trained Nurses' Club, 12, Buckingham Street, Strand, London, W.C., endorsing the envelope "Massage, to be forwarded."

(70) *Private Nursing* (*A. L.*)—There is only one mentioned in "How to Become a Nurse"—the "Margate Institute," Vicarage Crescent, Margate. Write to the lady superintendent.

(71) *Holidays* (*An Inquirer*).—Read the articles entitled "Holidays and Health" which appeared in The Hospital "Nursing Supplement" last summer between June and September. The Lakes were dealt with in the number for August 24th, 1895; North Wales in that for May 30th, 1896. Other articles will appear in the coming weeks, some of which we hope you may find useful. When you have settled upon your plans we shall be glad to help you in the matter of lodgings if it is in our power to do so.

(72) *Midwifery Training* (*Newcastle*).—Edinburgh Royal Maternity Hospital, Lauriston; Liverpool Lying-in Hospital and Ladies' Charity, Brownlow Hill; Manchester Maternity Hospital, 60, Upper Brook Street. For particulars of fees write direct to the matrons of the institutions.

(73) *Supervision* (*L. B. P.*)—Please explain your meaning more clearly. We have read your question several times and cannot at all make out what is the precise point upon which you wish for information.

(74) *Nursing Abroad* (*Nurse*).—Advertise and watch the advertisement columns in The Hospital "Nursing Supplement." The matron of the Kimberley Hospital was advertising for nurses the other day. Also you might write to the matrons of colonial and other hospitals; you will find names and addresses in Burdett's "Hospitals and Charities" (Scientific Press, 428, Strand, W.C.)

(75) *The Presentation Album* (*H.*).—Yes. The album may be seen at Mr. Albert Barker's, 5, New Bond Street, W., until the end of the month.

(76) *Medical Advice* (*Ipswich*).—Attached to the visiting staff of every large London hospital will be found the names of well known specialists. You will find these all given in Burdett's "Hospitals and Charities," and can make your own selection.

(77) *A Brass Plate* (*Mrs. J.*).—"Mrs. J., Trained Nurse, Visiting Nurse (Trained and Certificated)," we would suggest, and you might add any special particulars that seem desirable.

(78) *Holidays* (*L. C.*).—Read articles on "The Lakes" in The Hospital "Nursing Supplement" for August 24th, 1895, p. cxlv. Write to the Manager, Scientific Press, 428, Strand for the back number if you have not a copy. Be sure you take a tourist ticket, which enables you to break your journey when and where you like, and is cheaper.

### Nursing Exhibitions.

The following letter appeared in the *Times* for June 6th, signed "Justitia":—

"I notice that you describe the Nursing Exhibition which opened on Monday at St. Martin's Town Hall as 'the first of its kind which has been held.' In justice to others, will you kindly permit me to point out that the credit of having inaugurated an exhibition of this sort belongs rather to the committee of the Trained Nurses' Club, at whose premises, 12, Buckingham Street, Strand, a most interesting and successful exhibition of nursing appliances was opened on October 17th last year?"

## Figures and Tables

**Fig. 21. f1:**
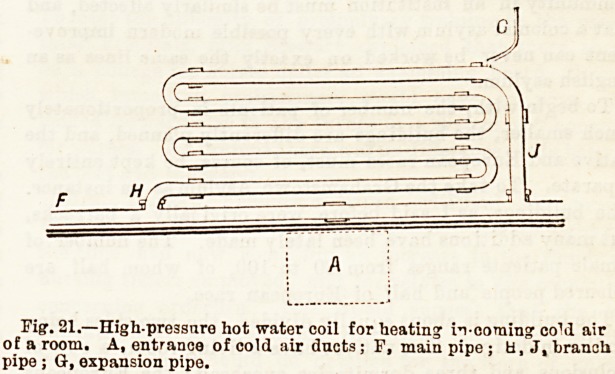
High-pressure hot water coil for heating in-coming cold air of a room. A, entrance of cold air ducts; F, main pipe; H, J, branch pipe; G, expansion pipe

**Fig. 22. f2:**